# Predictors of Psychologist Trainees’ Attitudes Toward and Interest in Future Work With Older Adult Clients

**DOI:** 10.1093/geroni/igaa057.1455

**Published:** 2020-12-16

**Authors:** Grace Caskie, Abigail Voelkner, MaryAnn Sutton

**Affiliations:** 1 Lehigh University, Bethlehem, Pennsylvania, United States; 2 Counseling Psychology, Bethlehem, Pennsylvania, United States

## Abstract

By 2035, 25% of the growing older adult population may be in need of mental health services (Novotney, 2018; Vespa, 2018). However, only a small proportion of psychologists currently identify as geropsychologists; thus, the number of geropsychologists will be insufficient to meet these future demands. Identifying variables that explain the variability in current psychology trainees’ expressed interest to engage in future clinical work with older adults is important so that training efforts can be targeted and the number of geropsychologists increased. Based on a multicultural framework and intergroup contact theory, this study examined contact with older adults, empathy, and multicultural competence as predictors of counseling and clinical psychology doctoral trainees’ attitudes toward and interest in working with older adult clients. A sample of 311 doctoral trainees (234 clinical PhD/PsyD, 78 counseling PhD/PsyD) were surveyed online. Structural equation modeling tested the hypothesized interrelationships between study variables. The model showed good fit to the data (χ2(82) = 179.803, p<.001, TLI=.93, CFI=.94, RMSEA=.06, SRMR=.06). Greater contact with older adults was significantly related to more positive attitudes about older adults and greater interest in working with older adults. More positive attitudes was significantly related to greater interest in working with older adults. Empathy was significantly related to more positive attitudes, but to less interest in working with this age group. Increasing the amount of contact experiences with older adults as part of doctoral training programs in counseling/clinical psychology may help to enhance trainees’ positive attitudes and interest in future clinical work with older adults.

